# The neuroprotective effects of human bone marrow mesenchymal stem cells are dose-dependent in TNBS colitis

**DOI:** 10.1186/s13287-017-0540-3

**Published:** 2017-04-18

**Authors:** Ainsley M. Robinson, Ahmed A. Rahman, Sarah Miller, Rhian Stavely, Samy Sakkal, Kulmira Nurgali

**Affiliations:** College of Health and Biomedicine, Victoria University, Melbourne, VIC Australia

**Keywords:** Inflammatory bowel disease, Intestinal inflammation, Mesenchymal stem cells, Enteric neurons, Dose-dependence

## Abstract

**Background:**

The incidence of inflammatory bowel diseases (IBD) is increasing worldwide with patients experiencing severe impacts on their quality of life. It is well accepted that intestinal inflammation associates with extensive damage to the enteric nervous system (ENS), which intrinsically innervates the gastrointestinal tract and regulates all gut functions. Hence, treatments targeting the enteric neurons are plausible for alleviating IBD and associated complications. Mesenchymal stem cells (MSCs) are gaining wide recognition as a potential therapy for many diseases due to their immunomodulatory and neuroprotective qualities. However, there is a large discrepancy regarding appropriate cell doses used in both clinical trials and experimental models of disease. We have previously demonstrated that human bone marrow MSCs exhibit neuroprotective and anti-inflammatory effects in a guinea-pig model of 2,4,6-trinitrobenzene-sulfonate (TNBS)-induced colitis; but an investigation into whether this response is dose-dependent has not been conducted.

**Methods:**

Hartley guinea-pigs were administered TNBS or sham treatment intra-rectally. Animals in the MSC treatment groups received either 1 × 10^5^, 1 × 10^6^ or 3 × 10^6^ MSCs by enema 3 hours after induction of colitis. Colon tissues were collected 72 hours after TNBS administration to assess the effects of MSC treatments on the level of inflammation and damage to the ENS by immunohistochemical and histological analyses.

**Results:**

MSCs administered at a low dose, 1 × 10^5^ cells, had little or no effect on the level of immune cell infiltrate and damage to the colonic innervation was similar to the TNBS group. Treatment with 1 × 10^6^ MSCs decreased the quantity of immune infiltrate and damage to nerve processes in the colonic wall, prevented myenteric neuronal loss and changes in neuronal subpopulations. Treatment with 3 × 10^6^ MSCs had similar effects to 1 × 10^6^ MSC treatments.

**Conclusions:**

The neuroprotective effect of MSCs in TNBS colitis is dose-dependent. Increasing doses higher than 1 × 10^6^ MSCs demonstrates no further therapeutic benefit than 1 × 10^6^ MSCs in preventing enteric neuropathy associated with intestinal inflammation. Furthermore, we have established an optimal dose of MSCs for future studies investigating intestinal inflammation, the enteric neurons and stem cell therapy in this model.

## Background

Crohn’s disease and ulcerative colitis are idiopathic inflammatory bowel diseases (IBD) characterized by chronic relapsing inflammation of the gastrointestinal (GI) tract [1]. Crohn’s disease manifests mostly in the ileum and colon, but can affect any region of the GI tract. Inflammation is discontinuous, focal and transmural progressing to the development of fistulas, abscesses, and strictures [2]. In contrast, ulcerative colitis is characterized by colonic mucosal inflammation extending proximally from the rectum [3]. Fundamental symptoms reported by IBD patients include abdominal pain, diarrhea, bloody stools, fecal urgency and rectal bleeding, as well as systemic symptoms of weight loss, fever, and fatigue [4, 5]. Furthermore, the risk of colorectal cancer increases as a complication of IBD [6].

The incidence of IBD is an evolving global concern, highest in westernized nations, such as Canada, Australia and countries in northern Europe, and increasing in developing nations in conjunction with their industrialization [7]. The peak age for IBD onset is 15 to 40 years and although not generally associated with mortality, the relentlessness of the disease negatively impacts on the patient’s quality of life [8]. Furthermore, both direct and indirect expenditures of IBD are substantial economic burdens on healthcare systems worldwide [9]. Conventional therapy for IBD includes anti-inflammatory drugs, corticosteroids, biologics, antimicrobial therapy and immunomodulators, which aim to treat symptoms rather than the underlying pathological mechanisms of the disease. Furthermore, these medications demonstrate long-term toxicity and/or failure to induce and maintain remission [10–12].

Abnormalities in the enteric nervous system (ENS) have been suggested to play a role in IBD pathogenesis for more than 50 years [13]. Comprising a complex network of neurons and glial cells embedded in the gut wall, the ENS intrinsically innervates the GI tract and is responsible for regulating and coordinating gut functions independently of the central nervous system [14]. Results from clinical and experimental models of IBD have reliably demonstrated enteric neuronal loss, axonal degeneration, glial cell hyperplasia, neuronal hyperexcitability and altered neurotransmission associated with intestinal inflammation [15–22]. Inflammation-induced changes to the neurochemical coding of myenteric neurons, specifically cholinergic and nitrergic subpopulations, disrupt GI functions and intestinal motility [23–25]. Furthermore, structural and functional alterations to the ENS persist beyond the resolution of active inflammation [26, 27]. These findings suggest that inflammation-induced insults to the ENS are integral in the generation of IBD symptoms and that the disease severity may be reduced via therapeutic strategies targeting the enteric neurons.

Due to unique therapeutic characteristics, mesenchymal stem cells (MSCs) have emerged as exciting candidates for cellular therapy against a range of immune-mediated and neurodegenerative disorders, including IBD [28–32]. Accumulating evidence has revealed that the protective mechanisms and endogenous regeneration initiated by MSCs are attributable to their capacity to produce and release an array of bioactive soluble factors acting in a paracrine manner to directly stimulate target cells and/or provoke nearby cells to emit functionally active mediators [33–35]. Further potential advantages of MSCs for cellular therapy include: in vitro expansion capacity, ease of isolation from adult tissue sources, low immunogenicity, capability for in vitro genetic modification and a safe and feasible profile for transplantation into humans [36–38].

While many studies report the effectiveness of MSC treatments in attenuating the mechanisms of disease, some MSC therapies are reported as being ineffective or only demonstrating short-term effectiveness [39–42]. Various factors, including cellular dose and timing of administration of MSCs, influence therapeutic efficacy of these cells [43]. Hence, it was suggested different doses of MSCs might have distinct immune or protective effects [44]. There is great variation among clinical trials and experimental models of disease in the injected dosage of MSCs [45, 46], suggesting that MSCs can effectively treat diseases in a dose-dependent manner [47–51]. In addition, defining an optimal MSC dose for both pre-clinical and clinical studies extends to benefits such as reduced production costs, less tissue required for MSC expansion, a lower chance of MSC mutation and a reduced likelihood of MSC accumulation in the filtering organs.

While it has been confirmed that MSCs migrate to sites of intestinal inflammation where they assist in the restoration and repair of the epithelial barrier and damaged tissue via anti-inflammatory actions [51–55], there are only a few studies examining the effects of MSC-based therapies in attenuating inflammation-induced enteric neuropathy [25, 35, 56, 57]. In these studies, it was concluded that locally applied bone marrow (BM)-MSCs administered at a dose of 1 × 10^6^ are neuroprotective towards enteric neurons compromised by 2,4,6-trinitrobenzene-sulfonate (TNBS)-induced inflammation [25, 35, 57]. These results provide the foundation for examining the neuroprotective potential of MSC therapy in intestinal inflammation. However, no studies have investigated the dose-response relationship of MSCs in protecting enteric neurons from damage and/or death induced by colitis. Therefore, the aim of this study was to investigate at which dose (1 × 10^5^, 1 × 10^6^ or 3 × 10^6^) human BM-MSCs are most beneficial in protecting and repairing enteric neurons following induction of colitis. This knowledge will define the optimal MSC dosage for treatment of enteric neuropathy associated with TNBS-induced inflammation in a guinea-pig model of colitis, as well as contribute towards future investigations into the mechanisms of MSC-stimulated enteric neuroprotection.

## Methods

### Animals

Male and female Hartley guinea-pigs weighing 140–280 g were obtained from South Australian Health and Medical Research Institute (SAHMRI, Adelaide, SA, Australia) and randomly assigned to experimental groups. All animals were housed in a temperature-controlled environment with 12-hour day/night cycles and had ad libitum access to food and water. All animal experiments in this study complied with the guidelines of the National Health and Medical Research Council (NHMRC) Australian Code of Practice for the Care and Use of Animals for Scientific Purposes under approval of the Victoria University Animal Experimentation Ethics Committee (approval number AEETH 12-012). All efforts were made to minimize animal suffering.

### Cell culture and passaging

MSCs derived from human BM-MSC cell lines BM-7025 and BM-7081 (Tulane University, New Orleans, LA, USA) were plated at an initial density of 60 cells/cm^2^ and incubated in complete culture medium (minimum essential medium (α-MEM) supplemented with 16.5% MSC-qualified fetal bovine serum (FBS), 100 U/mL penicillin/streptomycin, and 100X GlutaMAX) (all purchased from Gibco, Life Technologies, Mulgrave, VIC, Australia) at 37 °C. Expansion medium was replenished every 48–72 hours for 10–14 days until the cells were 70–85% confluent (maximum). MSCs were rinsed in 5 mL sterile phosphate-buffered solution (PBS) prior to incubation with 3 mL TrypLE Select (Gibco, Life Technologies, Mulgrave, VIC, Australia) for 3 minutes at 37 °C to detach cells. Enzymatic activity was neutralized by 8 mL of stop solution (α-MEM + 5% FBS) and MSCs were collected and centrifuged at 450 *g* for 5 minutes at room temperature. Cells were then resuspended in fresh culture medium and counted using a haemocytometer under a light microscope.

### MSC characterization

MSCs were cultured to the fourth passage for all experiments and characterized for their expression of surface antigens, differentiation potential, and colony-forming ability as previously described [25, 57]. All MSCs utilized in this study met criteria for defining in vitro human MSC cultures proposed by the International Society for Cellular Therapy (ISCT) [58].

### Induction of colitis

For the induction of colitis, TNBS (Sigma-Aldrich, Castle Hill, NSW, Australia) was dissolved in 30% ethanol to a concentration of 30 mg/kg and administered intra-rectally 7 cm proximal to the anus (total volume of 300 μL) by a lubricated silicone catheter [21]. For TNBS administration, guinea-pigs were anaesthetized with isoflurane (1–4% in O_2_) during the procedure. Sham-treated guinea-pigs underwent the same procedure without administration of TNBS.

### MSC treatments

Guinea-pigs in the MSC-treated groups were anaesthetized with isoflurane 3 hours after TNBS administration and administered MSC therapies by enema into the colon via a silicone catheter. MSCs were administered at a dose of 1 × 10^5^, 1 × 10^6^ or 3 × 10^6^ cells in 300 μL of sterile PBS. The peak of ethanol-induced epithelial damage occurs at 3 hours in TNBS-induced colitis [59], therefore this time point was selected for the administration of MSCs. Animals were held at an inverted angle following MSC treatments to prevent leakage from the rectum and were weighed and monitored daily following treatment. Guinea-pigs were culled via stunning and exsanguination 72 hours after TNBS administration [20]. Sections of the distal colon were collected for histological and immunohistochemical studies.

### Tissue preparation

Following dissection, tissues were immediately placed in oxygenated PBS (0.1 M, pH 7.2) containing an L-type Ca^2+^ channel blocker, nicardipine (3 μm) (Sigma-Aldrich, Castle Hill, NSW, Australia), to inhibit smooth muscle contraction. Tissues were cut open along the mesenteric border and then processed for whole-mount longitudinal muscle-myenteric plexus (LMMP) preparations and cross sections.

### LMMP preparations

Colon tissues were pinned flat with the mucosal side up and stretched to maximal capacity without tearing in a Sylgard-lined Petri dish. Tissues were fixed overnight at 4 °C in Zamboni’s fixative (2% formaldehyde and 0.2% picric acid) and subsequently washed for 3 × 10 minutes in dimethyl sulfoxide (DMSO) (Sigma-Aldrich, Castle Hill, NSW, Australia) followed by 3 × 10 minutes in 0.1 M PBS to remove fixative. Zamboni’s fixative was chosen for tissue fixation to minimize neural tissue autofluorescence. Distal colon samples were dissected to expose the myenteric plexus by removing the mucosa, submucosa and circular muscle layers prior to immunohistochemistry.

### Cross sections

Tissues for cross sections were pinned with the mucosal side up in a Sylgard-lined Petri dish, without stretching. Tissues for immunohistochemistry were fixed as described above and subsequently frozen in liquid nitrogen-cooled isopentane and optimum cutting temperature (OCT) compound (Tissue-Tek, Torrance, CA, USA). Samples were stored at -80 °C until they were cryosectioned (30 μm) onto glass slides for immunohistochemistry. Tissues for histology were fixed in 10% buffered formalin overnight at 4 °C and stored in 70% ethanol until paraffin embedding.

### Immunohistochemistry

Immunohistochemistry was performed on whole-mount LMMP preparations and cross sections of the distal colon as previously described [25, 35]. After a 1-hour incubation in 10% normal donkey serum (NDS) (Merck Millipore, Melbourne, VIC, Australia) diluted in 0.1 M PBS-0.1% Triton X-100 at room temperature, the samples were washed with 0.1 M PBS-0.1% Triton X-100 (2 × 5 minutes) and incubated with primary antibodies (Table 1) diluted in 2% NDS and 0.1 M PBS-0.1% Triton X-100 overnight at room temperature. Tissues were then washed in 0.1 M PBS-0.1% Triton X-100 (2 × 5 minutes) prior to incubation with secondary antibodies (Table 1) (diluted in 2% NDS and 0.1 M PBS-0.1% Triton X-100) for 2 hours at room temperature. Following 3 × 10 minutes washes in 0.1 M PBS-0.1% Triton X-100, LMMP preparations were mounted on glass slides with fluorescent mounting medium (DAKO, North Sydney, NSW, Australia).

**Table 1:** Antibodies used in this study

	Host species	Dilution	Supplier	Application in this study
Primary antibodies				
Anti-β-Tubulin class III	Rabbit	1:1000	Abcam, Melbourne, VIC, Australia	Cross sections
Anti-CD45 (clone IH-1)	Mouse	1:200	Abcam, Melbourne, VIC, Australia	Cross sections
Anti-choline acetyltransferase (ChAT)	Goat	1:500	Merck Millipore, Bayswater, Australia	LMMP preparations
Anti-Hu (clone 15A7.1)	Mouse	1:500	Merck Millipore, Bayswater, VIC, Australia	LMMP preparations
Anti-neuronal nitric oxide synthase (nNOS)	Goat	1:500	Novus Biologicals, Littleton, CO, USA	LMMP preparations
Anti-human leucocyte antigen (HLA)-A,B,C (conjugated to fluorescein isothiocyanate (FITC))	Human	1:50	BioLegend, San Diego, CA, USA	Cross sections
Secondary antibodies				
Alexa Fluor 594	Donkey anti-mouse	1:200	Jackson Immunoresearch Labs, West Grove, PA, USA	LMMP preparations
Alexa Fluor 594	Donkey anti-rabbit	1:200	Jackson Immunoresearch Labs, West Grove, PA, USA	Cross sections
FITC 488	Donkey anti-goat	1:200	Jackson Immunoresearch Labs, West Grove, PA, USA	LMMP preparations
FITC 488	Donkey anti-mouse	1:200	Jackson Immunoresearch Labs, West Grove, PA, USA	Cross sections

*LMMP* longitudinal muscle-myenteric plexus

### Histology

After fixation, tissues were paraffin embedded, sectioned at 5 μm, deparaffinized, cleared, and rehydrated in graded ethanol concentrations. For standard haematoxylin and eosin (H&E) staining, sections were immersed in xylene (3 × 4 minutes), 100% ethanol (3 minutes), 90% ethanol, (2 minutes), 70% ethanol (2 minutes), rinsed in tap water, haematoxylin (4 minutes), rinsed in tap water, Scott’s tap water (1 minute), eosin (3 minutes), rinsed in tap water, 100% ethanol (2 × 1 minute), xylene (2 × 3 minutes) and mounted on glass slides with distrene plasticizer xylene (DPX) mountant.

### Imaging

Confocal microscopy was undertaken on an Eclipse Ti confocal laser scanning system (Nikon, Tokyo, Japan). Fluorophores were visualized by using a 488 nm excitation filter for FITC and a 559 nm excitation filter for Alexa 594. Z-series images were acquired at a nominal thickness of 0.5 μm (512 × 512 pixels) with × 20 (dry, 0.75) or × 40 (oil immersion, 1.3) lenses. H&E-stained colon sections were visualized using an Olympus BX53 microscope (Olympus, Notting Hill, VIC, Australia) and images were captured with CellSense™ software.

### Quantitative analyses of immunohistochemical and histological data

In whole-mount LMMP preparations, the total number of myenteric neurons immunoreactive (IR) for Hu, neuronal nitric oxide synthase (nNOS) and choline acetyltransferase (ChAT) were counted within eight randomly captured images per preparation at × 20 magnification (total area 2 mm^2^), as well as per ganglia (average of ten ganglia per animal). Infiltration of leucocytes throughout the colon wall was assessed by counting the total number of CD45-IR cells within the mucosa and muscle layers in cross sections (total area 1.5 mm^2^). The density of nerve fibres was determined by measuring β-tubulin (III)-IR in eight randomly captured images at × 20 magnification. All images were captured under identical acquisition exposure time conditions and calibrated to standardized minimum baseline fluorescence. Images were converted from red, green, and blue (RGB) to grayscale 8 bit then to binary; changes in fluorescence from the baseline were measured using Image J software (National Institutes of Health, Bethesda, MD, USA). The area of immunoreactivity was then expressed as a percentage of the total area examined. Gross morphological damage in H&E-stained colon sections was assessed by histological grading of four parameters: mucosal flattening (0 = normal, 3 = severe flattening), occurrence of haemorrhagic sites (0 = none, 3 = frequent sites), loss of goblet cells (0 = normal, 3 = severe loss of cells) and variation of the circular muscle (0 = normal, 3 = considerable thickening of muscular layer) [25]. Quantitative analyses were conducted blindly.

### Statistical analysis

Statistical differences were determined by Student’s *t* test (two-tailed) or one-way ANOVA with Bonferroni post hoc test for multiple group comparisons using Prism v6.0 (Graphpad Software Inc., La Jolla, CA, USA). Data were considered statistically significant when *P* < 0.05. Data were presented as mean ± standard error of the mean (SEM), if not specified otherwise.

## Results

### MSCs migration and engraftment at the site of inflammation

**Fig. 1:**
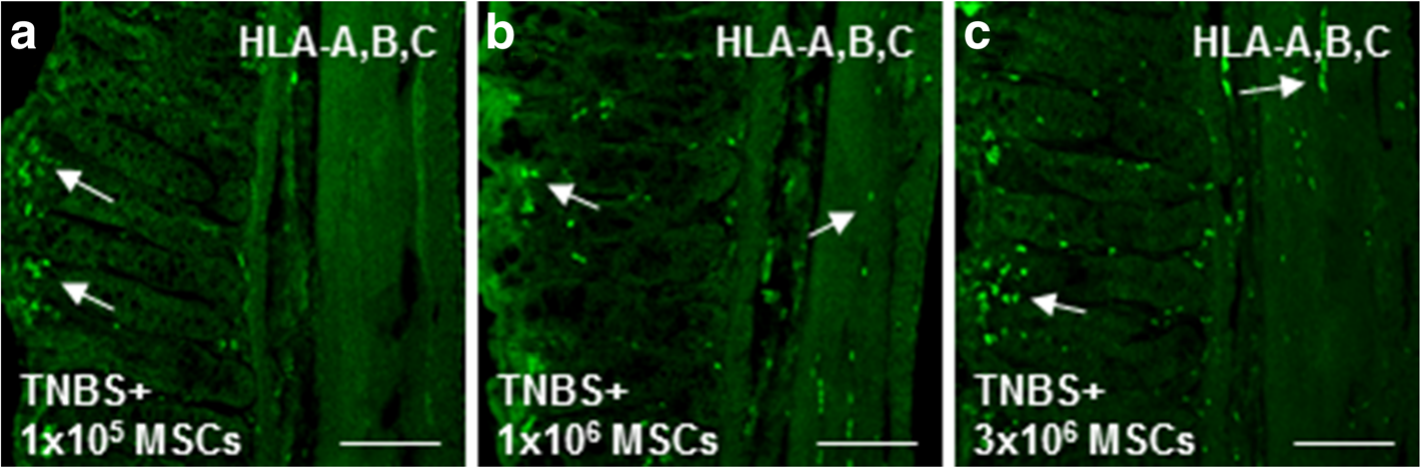
MSC homing within the inflamed colon. The migration and engraftment of MSCs (*white arrows*) to the site of TNBS-induced inflammation in sections of the guinea-pig colon was confirmed using anti-HLA-A,B,C antibody specific to human MHC class I at 72 hours post induction of colitis (**a**-**c**). Scale bars = 100 μm, *n* = 4/group/time point. *HLA-A,B,C* human leucocyte antigen, *IR* immunoreactive, *MSCs* mesenchymal stem cells, *TNBS* 2,4,6-trinitrobenzene sulfonic acid

The capacity of MSCs to migrate and engraft to the area of tissue damage and inflammation was assessed in sections of the distal colon from guinea-pigs treated with 1 × 10^5^, 1 × 10^6^ or 3 × 10^6^ MSCs administered by enema 3 hours after the induction of TNBS colitis. Transmural migration of MSCs within colon cross sections was identified by labelling with an antibody against human leucocyte antigen (HLA)-A,B,C, which detects major histocompatibility complex class I (MHC class I) antigens expressed by all human nucleated cells (Fig. 1). MSCs successfully engrafted into the intestinal wall evident by localization of HLA-A,B,C-positive cells in the colon sections collected at 72 hours post induction of colitis (Fig. 1a-c). HLA-A,B,C-positive cells were present mostly in the mucosal lamina propria in colon sections from guinea-pigs treated with 1 × 10^5^ MSCs (Fig. 1a). When administered at higher doses (1 × 10^6^ and 3 × 10^6^), transmural migration and engraftment of human MSCs into the colon wall to the level of the myenteric ganglia was evident (Fig. 1b-c).

### Effects of MSC treatment on tissue repair

**Fig. 2:**
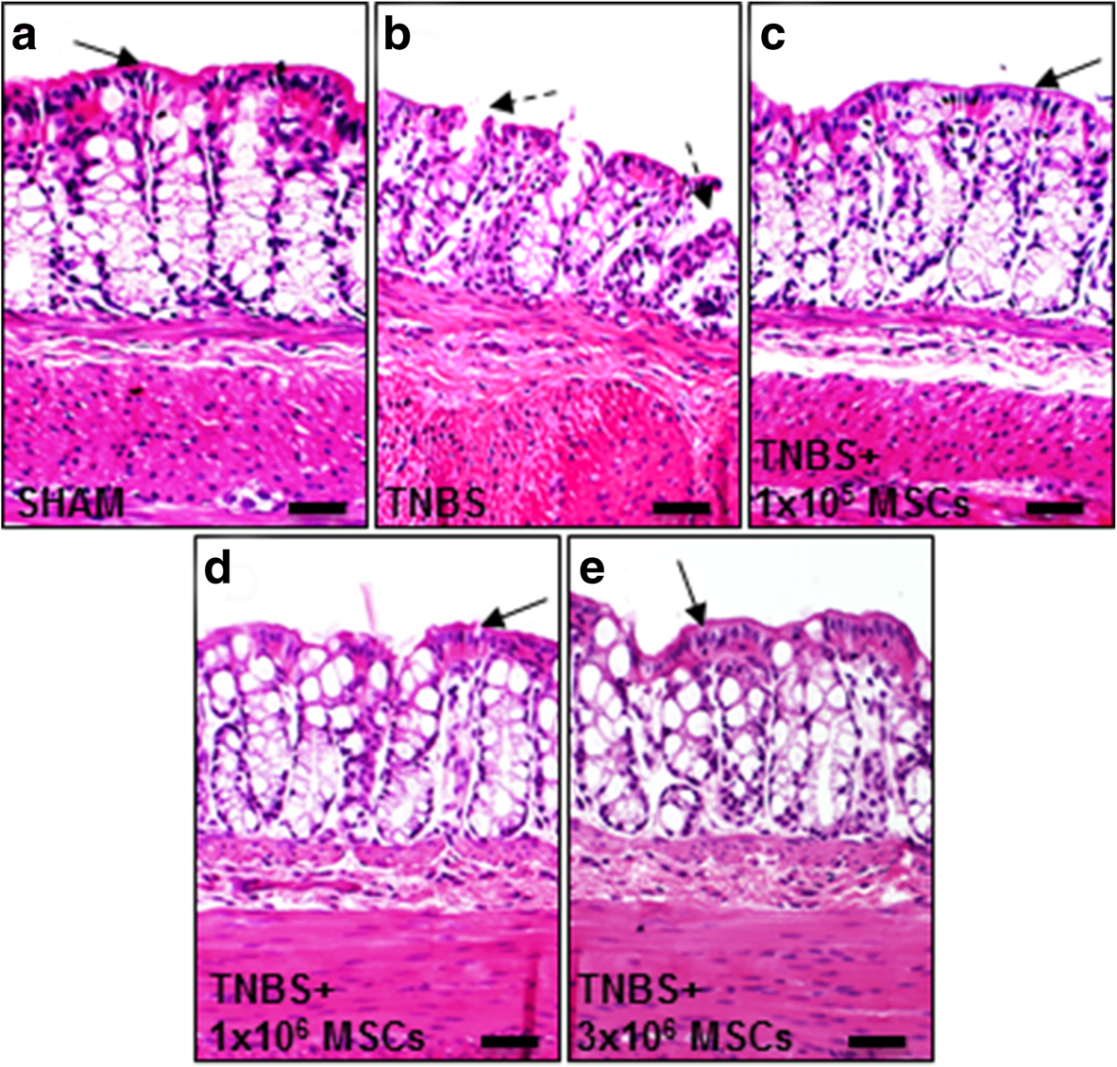
Gross morphological changes in the distal colon assessed in H&E-stained cross sections. A complete and continuous epithelial lining (*black arrows*) and regular arrangement of colonic layers was apparent in sections from sham-treated animals (**a**). Flattening of the glands, disruption to the epithelial lining (*dotted black arrows*) and goblet cell loss were evident in sections from TNBS-administered guinea-pigs at 72 hours post induction of colitis (**b**). H&E-stained sections from 1 × 10^5^, 1 × 10^6^ and 3 × 10^6^ MSC-treated animals revealed accelerated repair and restoration of the colonic architecture (**c**-**e**). Scale bars = 50 μm, *n* = 4/group/time point. *MSCs* mesenchymal stem cells, *TNBS* 2,4,6-trinitrobenzene sulfonic acid

Changes to the colonic architecture 72 hours after induction of TNBS-induced colitis were evaluated by gross morphological assessment of H&E-stained colon sections (Fig. 2). Continuous epithelial cell lining, regular structural arrangements of goblet cells and crypts and defined colonic layers were evident in H&E-stained colon cross sections from sham-treated guinea-pigs (histological score 0–1) (Fig. 2a). In contrast, sections from guinea-pigs in the TNBS-only group displayed disruptions to the epithelial lining, goblet cell loss, glandular distortion and flattening of crypts (histological score 2) (Fig. 2b). Sections from animals in all MSC-treated groups revealed accelerated healing of the mucosa and repair to levels comparable with sham-treated guinea-pigs (histological score 0–1 for all) (Fig. 2c-e).

### Dose-dependent effects of MSC treatments on leucocyte infiltration in the inflamed colon

**Fig. 3:**
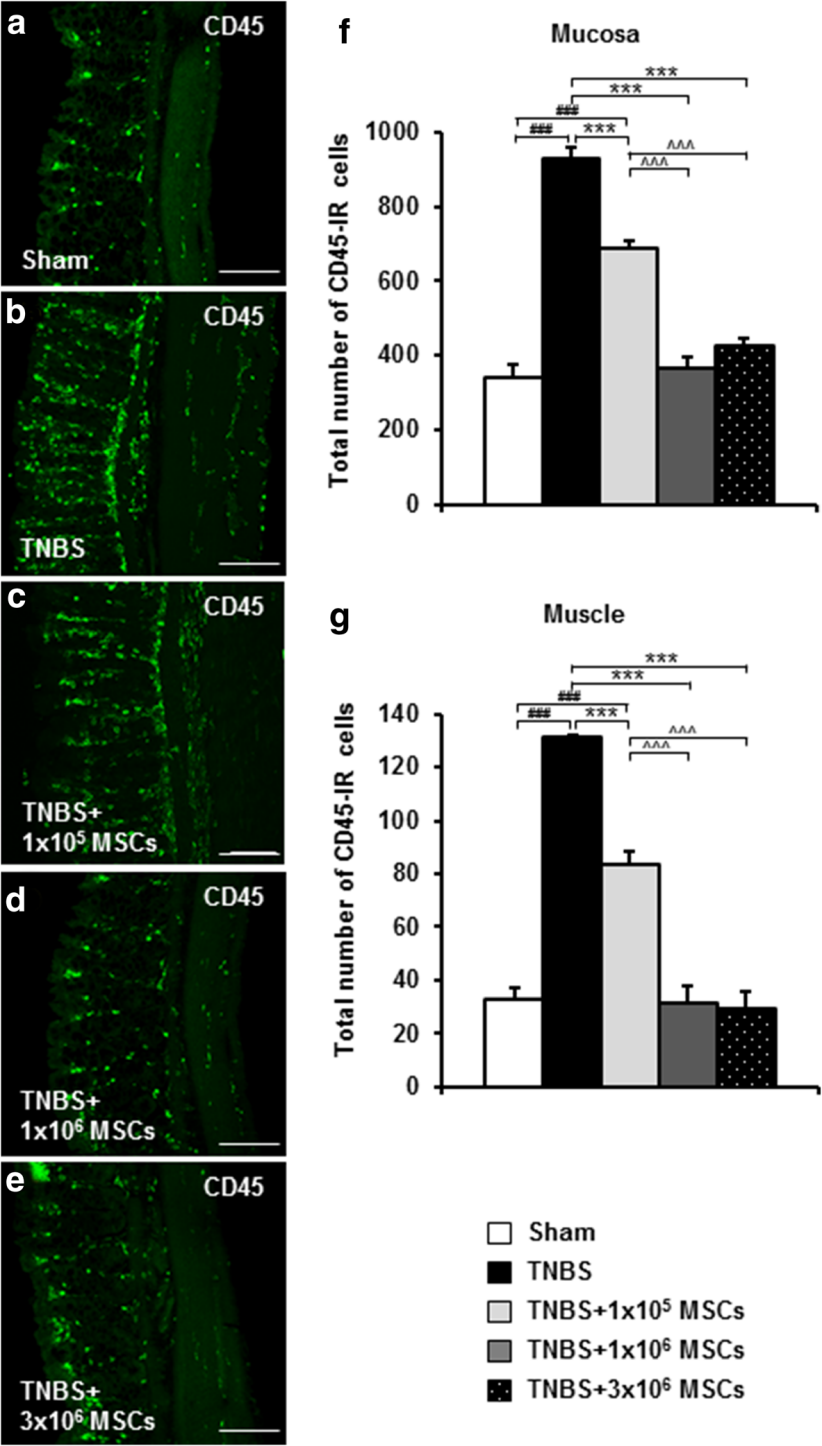
Leucocyte infiltration in colon cross sections. Sections of the guinea-pig distal colon were labelled with pan-leucocyte marker anti-CD45 to observe the effects of MSC treatments on leucocyte infiltration. CD45-IR leucocytes were visualized within the mucosa and muscular layers of distal colon sections from guinea-pigs collected at 72 hours post induction of colitis (**a**-**e**). Scale bars = 100 μm. The total number of CD45-IR cells per 1.5 mm^2^ area quantified in the mucosa (**f**) and muscular (**g**) layers of the colon cross sections. ^###^ *P* < 0.001 when compared to sham-treated guinea-pigs, ***P < 0.001 when compared to TNBS-only administered guinea-pigs, ^^^*P* < 0.001 when compared to 1 × 10^5^ MSC-treated guinea-pigs. *n* = 4/group/time point. *IR* immunoreactive, *MSCs* mesenchymal stem cells, *TNBS* 2,4,6-trinitrobenzene sulfonic acid

The severity of colitis and the anti-inflammatory effect of MSC treatments were assessed by quantitative analyses of CD45+ leucocytes in colon cross sections (Fig. 3). TNBS administration produced an increase in leucocytes within the mucosa and muscular layers at 72 hours when compared to sections from sham-treated guinea-pigs (mucosa: *P <* 0.001 and muscle: *P <* 0.001) (Table 2, Fig. 3a-b, f-g). The TNBS-induced increase in number of leucocytes in the mucosa and muscular layers was reduced by all MSC treatments compared to the TNBS-only group (*P <* 0.001 for all). However, leucocyte numbers in both the mucosa and muscular layers of 1 × 10^5^ MSC-treated animals were higher compared to sham-treated, 1 × 10^6^ MSC-treated and 3 × 10^6^ MSC-treated guinea-pigs (*P <* 0.001 for all) (Table 2, Fig. 3a-g).

**Table 2:** Dose-dependent effects of MSC treatment on leucocyte infiltration, nerve fibre density and number of myenteric neurons in the inflamed distal colon

	Sham	TNBS	TNBS + 1 × 10^5^ MSCs	TNBS + 1 × 10^6^ MSCs	TNBS + 3 × 10^6^ MSCs
Total no. CD45-IR cells					
Mucosa	341 ± 34	927 ± 31^###^	686 ± 23***^###^	368 ± 27***^^^	425 ± 25***^^^
Muscle	33 ± 4	131 ± 1^###^	83 ± 5***^###^	32 ± 6***^^^	29 ± 6***^^^
β-tubulin (III)-IR fibre density					
Mucosa	3.31 ± 0.09	1.68 ± 0.05^###^	2.54 ± 0.05***^###^	3.39 ± 0.08***^^^	3.35 ± 0.04***^^^
Muscle	5.54 ± 0.17	4.03 ± 0.11^###^	3.93 ± 0.07^###^	5.67 ± 0.10***^^^	5.50 ± 0.12***^^^
Total no. of Hu-IR myenteric neurons					
Ganglia	124 ± 2	108 ± 5^#^	104 ± 2^##^	128 ± 3**^^	127 ± 4**^^
Area	1498 ± 13	1330 ± 41^##^	1312 ± 20^###^	1510 ± 19***^^	1513 ± 12***^^^
Total no. of nNOS-IR myenteric neurons					
Ganglia	22 ± 1	27 ± 1^##^	27 ± 1^###^	20 ± 1***^^	22 ± 1**^^^
Area	262 ± 12	318 ± 7^###^	317 ± 5^###^	277 ± 6**^^	246 ± 3***^^^
Proportion of nNOS-IR myenteric neurons					
Ganglia	17 ± 1	26 ± 2^###^	27 ± 1^###^	16 ± 1***^^^	18 ± 1***^^^
Area	17 ± 1	24 ± 1^###^	24 ± 1^###^	18 ± 1***^^^	16 ± 1***^^^
Total no. of ChAT-IR myenteric neurons					
Ganglia	67 ± 1	52 ± 2^##^	57 ± 2^##^	64 ± 2**^	65 ± 3**^
Area	749 ± 29	545 ± 24^###^	585 ± 23^##^	708 ± 29**^	743 ± 14***^^
Proportion of ChAT-IR myenteric neurons					
Ganglia	54 ± 1	49 ± 1	55 ± 1	50 ± 1	52 ± 4
Area	50 ± 2	41 ± 1^##^	45 ± 2	47 ± 2*	49 ± 1*

*MSC* mesenchymal stem cell, *TNBS* 2,4,6-trinitrobenzene sulfonic acid, *IR* immunoreactive, *nNOS* neuronal nitric oxide synthase, *ChA*T Choline acetyltransferase

^#^ *P <* 0.05, ^##^ *P <* 0.01, ^###^ *P <* 0.001 when compared to sham-treated guinea-pigs

*P < 0.05, **P < 0.01, ***P < 0.001 when compared to TNBS-only administered guinea-pigs

^*P <* 0.05, ^^*P <* 0.01, ^^^*P <* 0.001 when compared to 1 × 10^5^ MSC-treated guinea-pigs

### Dose-dependent effects of MSC treatment on nerve fibre regrowth and enteric neuroprotection

**Fig. 4:**
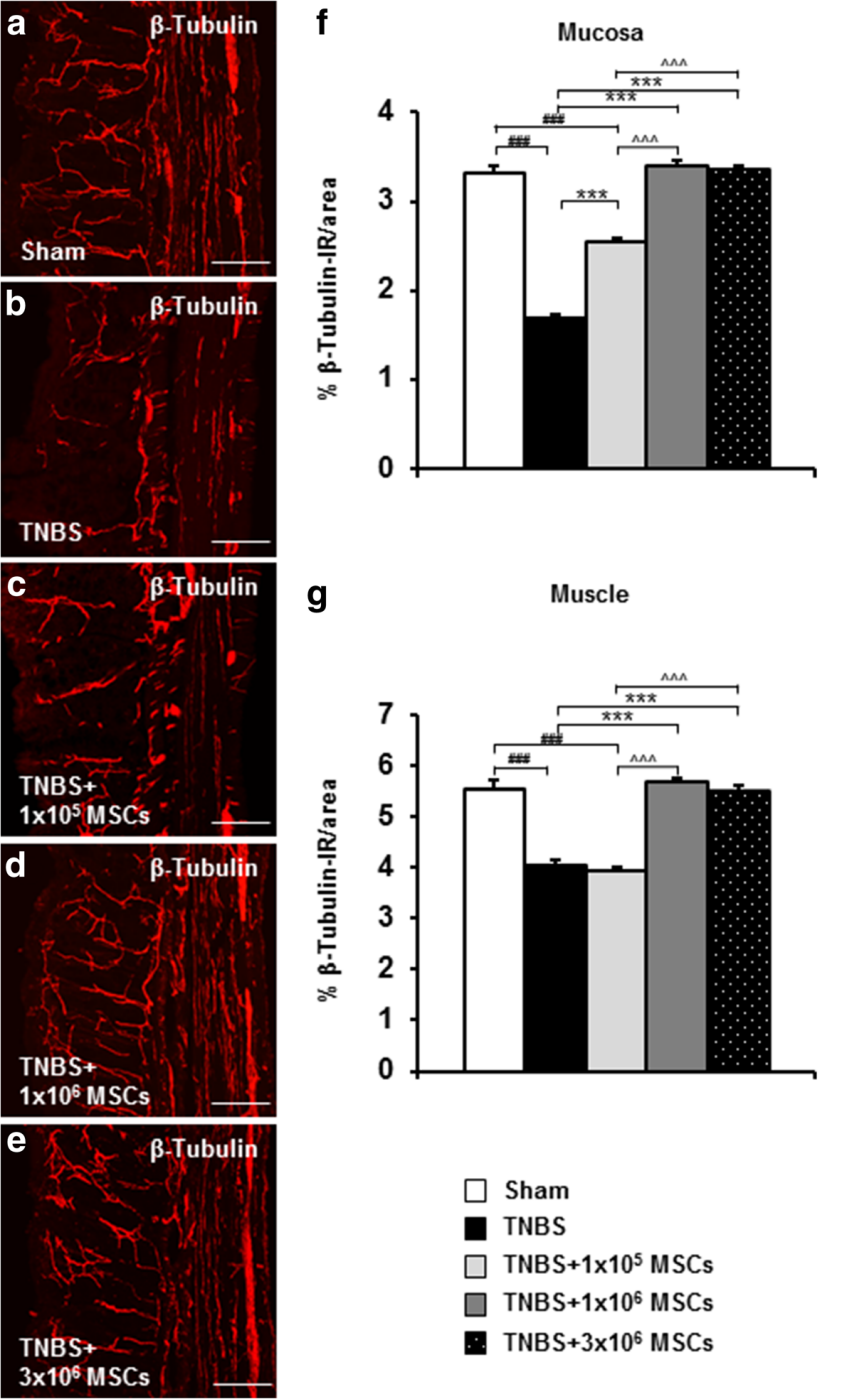
Nerve fibres in cross sections of the distal colon. Distribution of fibres labelled by neuron-specific anti-β-tubulin (III) antibody in colon sections from sham-treated, TNBS-only, 1 × 10^5^ MSC-treated, 1 × 10^6^ MSC-treated and 3 × 10^6^ MSC-treated guinea-pigs at 72 hours post induction of colitis (**a**-**e**). Scale bars = 100 μm. β-tubulin (III)-IR fibres were quantified in the mucosa (**f**) and muscular (**g**) layers of the colon. ^###^ *P* < 0.001 when compared to sham-treated guinea-pigs, ***P < 0.001 when compared to TNBS-only administered guinea-pigs, ^^^*P* < 0.001 when compared to 1 × 10^5^ MSC-treated guinea-pigs. *n* = 4/group/time point. *IR* immunoreactive, *MSCs* mesenchymal stem cells, *TNBS* 2,4,6-trinitrobenzene sulfonic acid

Nerve fibres innervating smooth muscles and mucosa were identified in cross sections of the guinea-pig distal colon by labelling with an antibody specific to neuronal microtubule protein β-tubulin (III) (Fig. 4). Regularly distributed β-tubulin (III)-IR fibres were observed within the mucosal gland cores, submucosal and muscular layers of colon sections from sham-treated guinea-pigs (Fig. 4a). Following TNBS administration, β-tubulin (III)-IR fibres within the mucosa were disordered, patchy, and arranged irregularly (Fig. 4b). Quantitative analysis confirmed a reduction in β-tubulin (III)-IR fibre density in both mucosa (*P <* 0.001) and muscle (*P <* 0.001) layers of the colon sections from TNBS-administered guinea-pigs when compared to sections from sham-treated animals (Table 2, Fig. 4f-g). Treatment with 1 × 10^5^ MSCs improved the nerve fibre density in the mucosa (*P <* 0.001) compared to the TNBS-only administered group. However, the density of fibres in both the mucosa and muscle was still lower compared to the sham-treated group (*P <* 0.001 for both) (Table 2, Fig. 4c, f–g). In contrary, the morphology of β-tubulin (III)-IR fibres in mucosal and muscular layers of colon sections from guinea-pigs treated with 1 × 10^6^ and 3 × 10^6^ MSCs were comparable to those in sections from sham-treated animals (Figs. 4d-e). When quantified, β-tubulin (III)-IR fibre density in mucosa and muscles in colon sections from 1 × 10^6^ and 3 × 10^6^ MSC-treated guinea-pigs was higher compared to both TNBS-only and 1 × 10^5^ MSC-treated guinea-pigs (*P <* 0.001 for all, Fig. 4f–g).

**Fig. 5:**
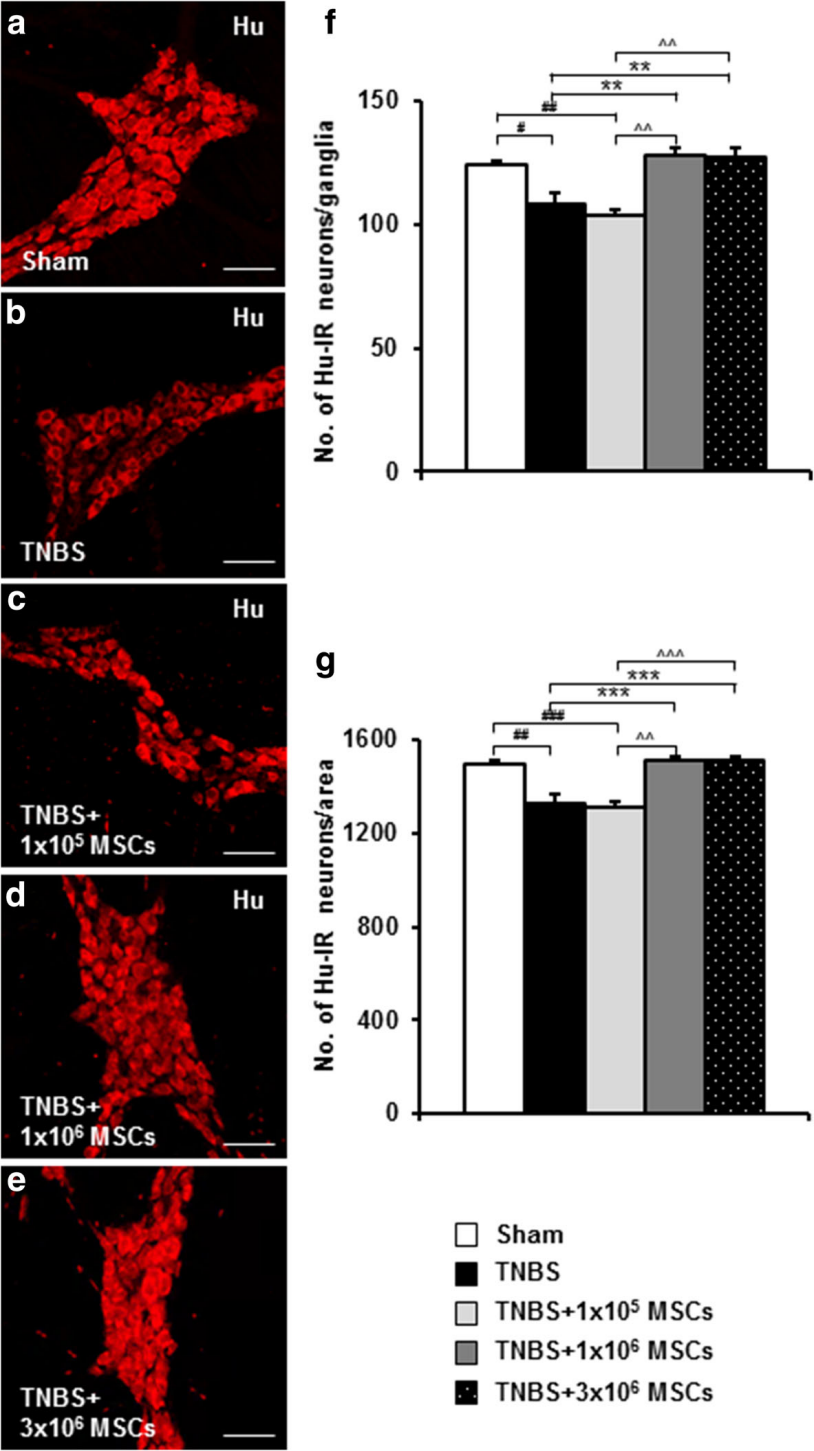
Effects of MSC treatments on the total number of myenteric neurons. Myenteric neurons were identified by anti-Hu antibody in whole-mount LMMP preparations of the distal colon from sham-treated, TNBS-only, 1 × 10^5^ MSC-treated, 1 × 10^6^ MSC-treated and 3 × 10^6^ MSC-treated guinea-pigs 72 hours post induction of colitis (**a**-**e**). Scale bars = 100 μm. The total number of Hu-IR neurons were counted per ganglion (average of ten ganglia) (**f**) and per 2 mm^2^ area (**g**) of the colon. ^#^ *P <* 0.05, ^##^ *P <* 0.01, ^###^ *P* < 0.001 when compared to sham-treated guinea-pigs, **P < 0.01, ***P < 0.001 when compared to TNBS-only administered guinea-pigs, ^^*P <* 0.01, ^^^*P* < 0.001 when compared to 1 × 10^5^ MSC-treated guinea-pigs. *n* = 4/group/time point. *IR* immunoreactive, *LMMP* longitudinal muscle-myenteric plexus, *MSCs* mesenchymal stem cells, *TNBS* 2,4,6-trinitrobenzene sulfonic acid

To investigate whether 1 × 10^5^, 1 × 10^6^ and 3 × 10^6^ MSC treatments were effective in preventing loss of myenteric neurons, neuronal cell bodies were labelled with the pan-neuronal marker anti-Hu antibody in whole-mount LMMP preparations of the distal colon (Fig. 5). The number of Hu-IR neurons was counted per ganglion and per 2 mm^2^ area. The number of Hu-IR myenteric neurons was decreased in colon preparations from TNBS-only guinea-pigs compared to sham-treated animals (ganglia: *P <* 0.05; area: *P <* 0.01) (Table 2, Fig. 5a-b, f-g). Treatment with 1 × 10^5^ MSCs did not prevent myenteric neuronal loss associated with colitis (Table 2, Fig. 5c, f-g). However, treatments with 1 × 10^6^ and 3 × 10^6^ MSCs attenuated neuronal loss associated with TNBS-induced inflammation (ganglia: *P <* 0.01 for both; area: *P <* 0.001 for both) compared to the TNBS group (Table 2, Fig. 5d-e, f-g).

### Dose-dependent effects of MSC treatments on inhibitory and excitatory myenteric neurons

**Fig. 6:**
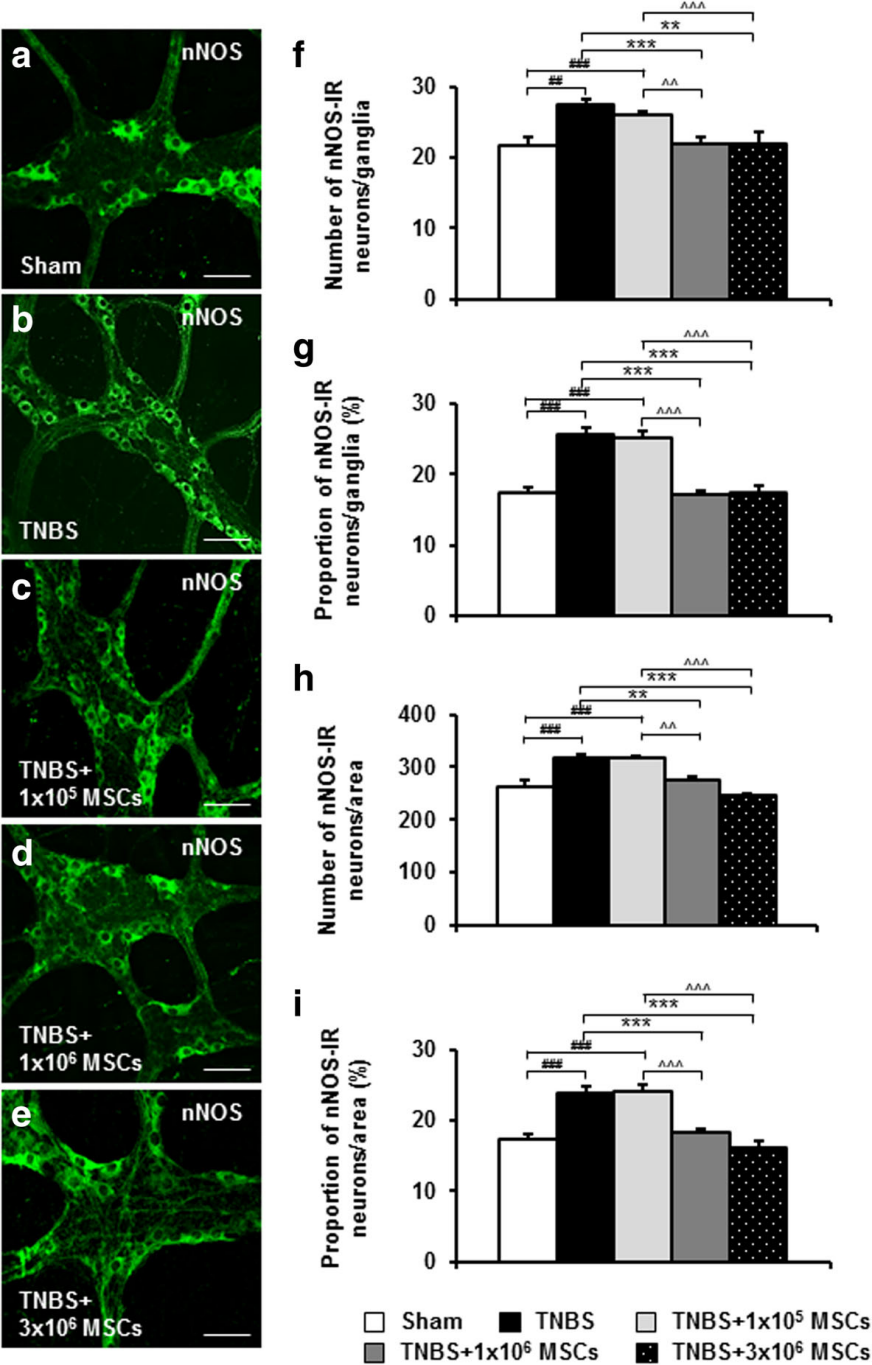
Effects of MSC treatments on nNOS-IR myenteric neurons. Inhibitory myenteric neurons were identified by anti-nNOS antibody in whole-mount LMMP preparations of the distal colon from sham-treated, TNBS-only, 1 × 10^5^ MSC-treated, 1 × 10^6^ MSC-treated and 3 × 10^6^ MSC-treated guinea-pigs at 72 hours post induction of colitis (**a**-**e**). Scale bars = 100 μm. The total number of nNOS-IR neurons was counted per ganglion (average of ten ganglia) (**f**) and per 2 mm^2^ area (**h**) of the colon. The proportion of nNOS-IR neurons to Hu-IR neurons per ganglia (**g**) and per 2 mm^2^ area (**i**). ^##^ *P <* 0.01, ^###^ *P* < 0.001 when compared to sham-treated guinea-pigs, **P < 0.01, ***P < 0.001 when compared to TNBS-only administered guinea-pigs, ^^*P <* 0.01, ^^^*P* < 0.001 when compared to 1 × 10^5^ MSC-treated guinea-pigs. *n* = 4/group/time point. *LMMP* longitudinal muscle-myenteric plexus, *nNOS* neuronal nitric oxide synthase, *IR* immunoreactive, *MSCs* mesenchymal stem cells, *TNBS* 2,4,6-trinitrobenzene sulfonic acid

Changes in inhibitory and excitatory myenteric muscle motor and interneurons underlie inflammation-induced colonic dysmotility, therefore we investigated the effects of MSC-based therapies on these two major subpopulations of neurons. Inhibitory neurons were labelled with anti-nNOS antibody in whole-mount LMMP preparations of the distal colon (Fig. 6). The number of nNOS-IR neurons per ganglion and per area was increased in the myenteric plexus from the TNBS-only group compared with sham-treated animals (ganglia: *P <* 0.01; area: *P <* 0.001). The proportion of nNOS-IR neurons to the total number of Hu-IR neurons was increased in TNBS-only guinea-pigs compared with sham-treated (*P <* 0.001 for all) (Table 2, Fig. 6a-b, f-i). The number and proportion of nNOS-IR neurons were comparable between TNBS-only administered guinea-pigs and 1 × 10^5^ MSC-treated animals (Table 2, Fig. 6c). Thus, nNOS-IR neurons were elevated in preparations from 1 × 10^5^ MSC-treated animals compared to sham-treated (*P <* 0.001 for both ganglia and area), 1 × 10^6^ MSC-treated (*P <* 0.01 for both ganglia and area) and 3 × 10^6^ MSC-treated (*P <* 0.001 for both ganglia and area) guinea-pigs (Table 2, Fig. 6f-i). Correspondingly, the proportion of nNOS-IR neurons was also increased (*P <* 0.001 for all). Both 1 × 10^6^ and 3 × 10^6^ MSC treatments prevented the increase in the total number (1 × 10^6^ - ganglia: *P <* 0.001; area: *P <* 0.01; 3 × 10^6^ - ganglia: *P <* 0.01; area: *P <* 0.001) and proportion (*P <* 0.001 for all) of nNOS-IR neurons compared to the TNBS-only group (Table 2, Fig. 6d-e, f-i).

**Fig. 7:**
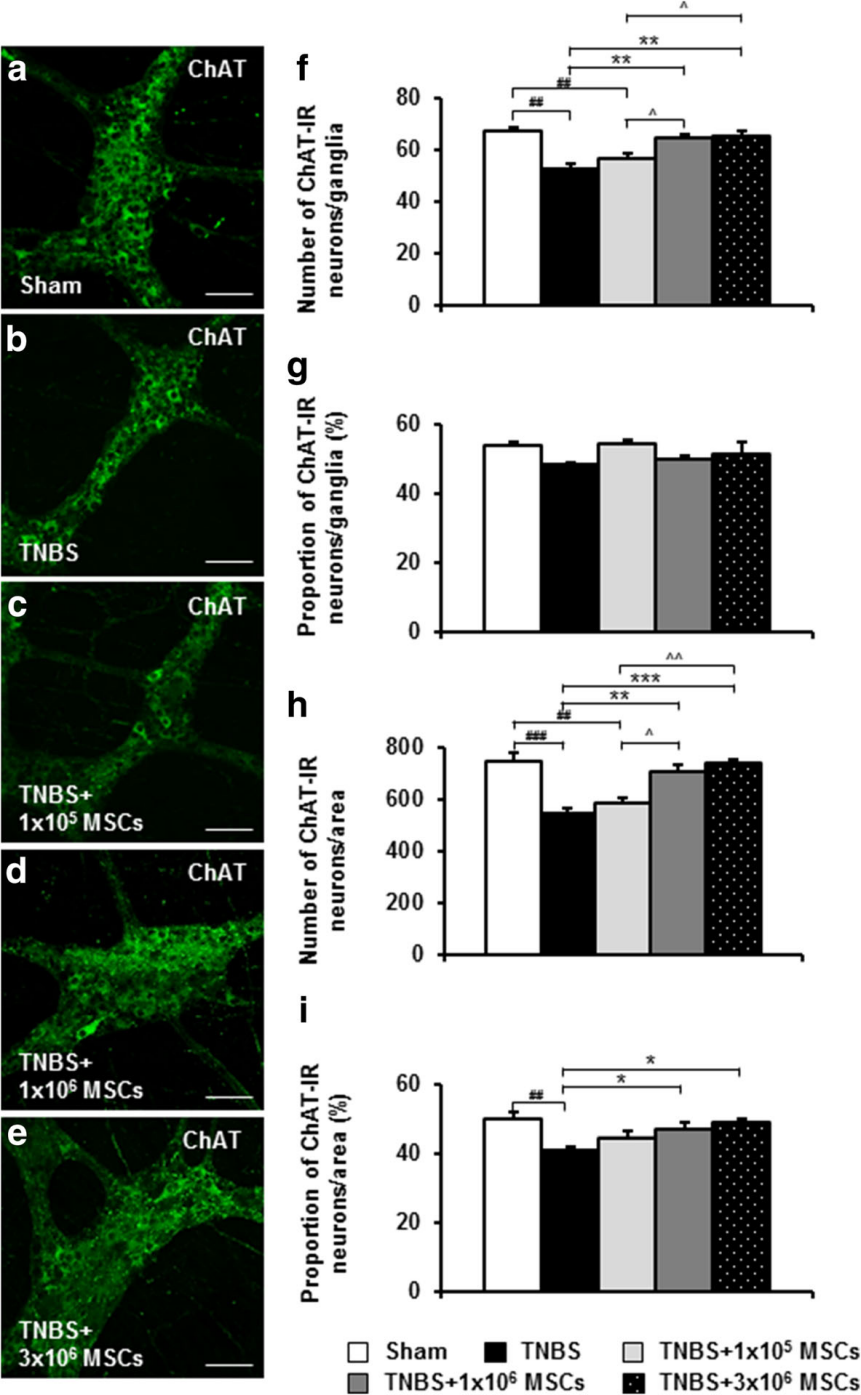
Effects of MSC treatments on ChAT-IR myenteric neurons. Excitatory myenteric neurons were identified by anti-ChAT antibody in whole-mount preparations of the distal colon from sham-treated, TNBS-only, 1 × 10^5^ MSC-treated, 1 × 10^6^ MSC-treated and 3 × 10^6^ MSC-treated guinea-pigs (**a**-**e**). Scale bars = 100 μm. The total number of ChAT-IR neurons were counted per ganglion (average of ten ganglia) (**f**) and per 2 mm^2^ area (**h**) of the colon. The proportion of ChAT-IR neurons to Hu-IR neurons per ganglia (**g**) and per area (**i**). ^##^ *P <* 0.01, ^###^ *P* < 0.001 when compared to sham-treated guinea-pigs, **P < 0.01, ***P < 0.001 when compared to TNBS-only administered guinea-pigs, ^*P <* 0.05, ^^*P* < 0.01 when compared to 1 × 10^5^ MSC-treated guinea-pigs. *n* = 4/group/time point. *ChAT* choline acetyltransferase, *IR* immunoreactive, *MSCs* mesenchymal stem cells, *TNBS* 2,4,6-trinitrobenzene sulfonic acid

Excitatory muscle motor and interneurons were identified using ChAT immunoreactivity in whole-mount preparations of the distal colon (Fig. 7). Quantification of ChAT-IR neurons revealed a decrease in the TNBS-only group compared to sham-treated guinea-pigs (ganglia: *P <* 0.01; area: *P <* 0.001) (Table 2, Fig. 7a-b, f, h). The number of ChAT-IR neurons in preparations from 1 × 10^5^ MSC-treated guinea- pigs was comparable to the TNBS-only group, indicating that this dose was not effective in preventing the TNBS-induced decrease in ChAT-IR neurons (Table 2, Fig. 7c, f, h). ChAT-IR neurons were reduced in 1 × 10^5^ MSC-treated animals compared to sham-treated (*P <* 0.01 for both ganglia and area), 1 × 10^6^ MSC-treated (*P <* 0.05 for both ganglia and area) and 3 × 10^6^ MSC-treated (ganglia: *P <* 0.05; area: *P <* 0.01) guinea-pigs. Treatment with 1 × 10^6^ MSCs (*P <* 0.01 for both ganglia and area) and 3 × 10^6^ MSCs (ganglia: *P <* 0.01; area: *P <* 0.001) prevented the TNBS-induced loss of ChAT-IR neurons with numbers comparable to the sham-treated group (Table 2, Fig. 7d-e, f, h). When quantified per ganglia, there were no differences between any groups in the proportion of ChAT-IR neurons to total number of neurons (Fig. 7g). However, when quantified per area (Fig. 7i), the proportion of ChAT-IR neurons to Hu-IR neurons was reduced in TNBS-only administered animals when compared to sham-treated (*P <* 0.01), 1 × 10^6^ MSC-treated (*P <* 0.05) and 3 × 10^6^ MSC-treated (*P <* 0.05) guinea-pigs.

## Discussion

In this study, we compared different doses of human BM-MSCs for their neuroprotective efficacy in a guinea-pig model of TNBS-induced colitis. Both 1 × 10^6^ and 3 × 10^6^ MSC treatments demonstrated therapeutic efficacy in the accelerated repair of colonic architecture, reduced leucocyte infiltration transmurally through the colon wall, regeneration of nerve fibres, and were equally neuroprotective in the amelioration of myenteric neuronal loss and changes to the neurochemical coding of their subpopulations. When administered at a dose of 1 × 10^5^, BM-MSCs offered some therapeutic benefit in healing of the colonic architecture, protection of nerve fibres and the offset of CD45-IR cells in the mucosa, however, were less effective in the attenuation of neuropathy at the level of the myenteric plexus. Thus, a dose of 1 × 10^6^ MSCs is necessary to ameliorate the effects of TNBS-induced inflammation at the level of the myenteric plexus; further increases in dose provide consistent efficacy without promotion of benefit.

In IBD patients with fistulae and luminal inflammation it has been demonstrated that therapy with both BM and adipose MSCs is safe, feasible and efficacious [60–64]. However, there is inconsistency regarding the most effective dose; some studies report reduced disease activity and fistula closure with doses ranging from 3 × 10^6^–60 × 10^6^ MSCs [60, 61, 65, 66]. Additionally, some studies have reported positive outcomes with dose regimes based relative to fistula size (dose range 1 × 10^6^–4 × 10^7^ cells/cm length of fistula (average number of injected cells: 20 × 10^6^–15.8 × 10^7^)) [63, 67, 68] or patient bodyweight (1 × 10^6^–2.7 × 10^6^ cells/kg) [62, 64]. In experimental models of colitis, BM-MSCs derived from rats [52–54, 69–71], mice [55, 72–74], guinea-pigs [56] and humans [25, 35, 56, 57, 75, 76] have been investigated for therapeutic efficacy. In addition, some studies have assessed adipose MSCs derived from these species [51, 56, 70, 77–80], as well as human umbilical cord, umbilical cord blood and gingiva [75, 81–83]. Overall, intravenous, intraperitoneal and local administration of MSCs from various sources and species have been reported to ameliorate experimental colitis however, similarly to clinical trials, there is no consistency regarding the most efficacious dose. Studies report therapeutic efficacy in ameliorating colitis following MSC application with doses of 2 × 10^3^ [69], 2 × 10^4^ [54], 5 × 10^5^ [71], 0.5 × 10^6^ [76], 1 × 10^6^ [25, 35, 51, 55–57, 72–74, 76, 78, 79, 81, 83], 2 × 10^6^ [70, 75, 80, 82], 5 × 10^6^ [51, 53], 1 × 10^7^ [52, 77] MSCs.

In this study, we employed MSCs derived from human BM. Human MSCs are the most characterized, clinically applied as a potential regenerative cell therapy [84] and defined based upon three minimal criteria (i) plastic adherence, (ii) trilineage differentiation, (iii) surface expression of CD73, CD90, CD105 and absence of expression of CD45, CD34, CD14 or CD11b, CD79α or CD19, and human leucocyte antigen (HLA)-DR [58]. MSCs used in this study were validated according to these guidelines issued by the International Society for Cellular Therapy. On the other hand, MSCs from animal origin have been defined as cells that fulfil the first two criteria [45]. Comparison of animal and human-derived MSCs has revealed a high degree of concordance [85–87]. However, differences have been reported in genomic stability [88, 89], differentiation potential [90], surface antigen expression and immunoregulatory capabilities [91] making it difficult to directly extrapolate the results obtained on animal MSCs to human MSCs. MSCs were originally derived from the BM, are the most frequently investigated cell type [92] and are often designated as the gold standard in the treatment of various inflammatory conditions [93–96]. Furthermore, human BM-MSCs are the most therapeutic in the treatment of enteric neuropathy and plexitis associated with TNBS-induced colitis [57].

Of particular relevance to the therapeutic application of MSCs is their fate post-implantation. Ambiguity seen in the efficacy of MSCs, in both animal studies and clinical trials, with therapies being ineffective or only temporarily effective could be due to suboptimal application of MSCs. Previous studies have indicated MSC efficacy may be affected by the timing of delivery [28] and administration during the earlier phases of inflammation is favourable for therapeutic results [97–99]. Therefore, in consistency with our previous studies, BM-MSCs were administered 3 hours after TNBS; the time point when substantial mucosal damage occurs [59].

The migratory and homing capacity of MSCs is facilitated by their expression of a wide array of chemokine receptors and adhesion molecules that respond to chemoattractant signals released from host cells at the site of injury [97]. MHC class I molecules are expressed on the surface of viable human MSCs promoting immune rejection and assisting in engraftment into damaged tissue via the absence of co-stimulatory ligands/receptors and release of immunosuppressive factors [45, 100, 101]. MSC migration to the area of inflammation and subsequent engraftment into the damaged tissue is an inaugural part of the tissue repair/regeneration process and indispensable for therapeutic efficacy. In this study, we labelled sections of the guinea-pig colon with anti-HLA-A,B,C antibody to evaluate the successful migration and engraftment of MSCs within the inflamed intestinal wall. MSCs engrafted into the mucosa at the initial site of TNBS-induced inflammation in all MSC-treated groups. However, in sections from guinea-pigs administered 1 × 10^6^ or 3 × 10^6^ MSCs, HLA-A,B,C-positive cells were observed at the level of the myenteric plexus in addition to the mucosal layer. The successful migration and engraftment of enema-applied MSCs into the inflamed colonic wall observed in our study is consistent with previous reports demonstrating implantation of locally administered MSCs into target tissues, especially in inflammatory conditions [52, 102]. Subsequently, the outcomes of the treatment were more pronounced in animals treated with 1 × 10^6^ and 3 × 10^6^ MSCs compared to those treated with 1 × 10^5^ MSCs.

Reduced disease activity, endoscopic and histopathologic severity of colitis, and infiltration of neutrophils into the colon are commonly evaluated to determine the effectiveness of MSC treatments in both clinical trials and experimental models of IBD. Within these parameters examining the therapeutic efficacy of various MSC doses at the level of the mucosa only, we could conclude that BM-MSCs ameliorate experimental colitis at a dose as low as 1 × 10^5^ MSCs. However, previous studies have reported marked structural and functional changes to the ENS in IBD accompanied by infiltration of inflammatory cells to the submucosa and myenteric plexus [103–105]. Alterations to the ENS persist long after resolution of acute intestinal inflammation reflected through changes in gut function, colonic dysmotility, hypersensitivity and dysfunction [104, 106], and myenteric plexitis has been shown to be predictive of IBD recurrence [103, 105]. Therefore, we further investigated the therapeutic efficacy of varying doses of BM-MSCs at the level of the myenteric plexus.

In this study, MSCs were effective in reducing leucocyte infiltration to the myenteric plexus when administered at doses of 1 × 10^6^ and 3 × 10^6^, but not at a dose of 1 × 10^5^. It may be proposed that while some immunomodulatory effect is occurring following application of 1 × 10^5^ MSCs, it is not strong enough to combat all inflammation since leucocyte numbers were reduced at the mucosal level in this group, but not at the myenteric level. While the immunomodulatory mechanisms of MSCs have not been completely elaborated, it is known that in order for MSCs to wield their immunosuppressive capacities, they must be induced by inflammatory cytokines within a pro-inflammatory microenvironment [107]. The increased numbers of leucocytes in the colonic wall following induction of TNBS colitis provided sufficient pro-inflammatory stimuli for activation of MSCs. Hence, the weaker influence demonstrated by the lower dose of MSCs suggests that the anti-inflammatory effect was hindered by a smaller quantity of MSCs rather than their immunomodulatory capacity. This is reflected by localization of MSCs within the inflamed colon where HLA-A,B,C-positive cells were evident in the mucosa only in sections from 1 × 10^5^ MSC-treated animals.

It is generally considered that the anti-inflammatory properties of MSCs function via direct interaction with target cells and/or production of diverse soluble factors [34, 108]. Many of the MSC-associated biological effects are mediated by paracrine mechanisms engaging the release of cytokines, chemokines and growth factors [34, 109, 110] and may be exerted by the induction and stimulation of endogenous host progenitor cells to improve the regenerative process [72, 111]. In animal models of colitis, MSC application efficiently reduces T helper 1 and T helper 17 responses and downregulates pro-inflammatory cytokines (such as tumour necrosis factor alpha (TNF-α), interleukin (IL)-1β, IL-6, IL-17, inducible nitric oxide synthase (iNOS), cyclooxygenase-2 (COX-2) and interferon gamma (IFN-γ) while enhancing the numbers of regulatory T cells and upregulating anti-inflammatory cytokines (such as IL-10) [112–115]. The proportion of mucosal and peripheral regulatory T cells was also increased after MSC treatment of CD fistulae [63]. These findings suggest that the paracrine actions of MSCs have an anti-inflammatory affect in IBD associated with inhibition of nuclear factor kappa B (NF-κB) signalling pathways. Furthermore, paracrine actions of MSCs have be shown to diminish free radicals and impede oxidative stress, prevent apoptosis via the extrinsic death receptor signal pathway and the intrinsic mitochondrial signal pathway and stimulate endogenous mechanisms of intestinal epithelial repair [111, 112].

It remains unclear whether changes to the ENS are the cause or the consequence of inflammation; however, in this study TNBS-induced plexitis was associated with damage to nerve fibres and loss of myenteric neurons, as well as changes in their subpopulations. Similar to the limited anti-inflammatory effect discussed above, sections from animals treated with 1 × 10^5^ MSCs revealed some nerve fibre regrowth, but not to the level of sham-treated animals. In contrast, significant regeneration and regrowth of nerve fibres in the colon were associated with 1 × 10^6^ and 3 × 10^6^ MSC treatments in this study. MSCs improve axonal and nerve regeneration through the production of local neurotrophic factors for induction of axonal growth, including brain-derived neurotrophic factor, nerve growth factor and insulin-like growth factor-1 [116, 117]. Hence, differences in the level and areas of MSC engraftment demonstrated in sections from 1 × 10^5^ MSC-treated animals compared to 1 × 10^6^ and 3 × 10^6^ MSC-treated guinea-pigs maybe associated with a reduction in the expression of neurotrophic factors. However, this needs to be further investigated.

Our study demonstrated a persistent loss of myenteric neurons to be associated with TNBS-induced inflammation in the distal colon of guinea-pigs 72 hours after induction of colitis. Consistent with our findings, the quantity of myenteric neurons was found to be reduced in the guinea-pig intestine subsequent to intra-rectal administration of TNBS in previous studies [16, 21, 25, 35, 56, 57]. In this study, neuronal loss was not prevented following treatment with 1 × 10^5^ MSCs and the number of myenteric neurons was comparable to the TNBS-only administered group. On the other hand, doses of 1 × 10^6^ and 3 × 10^6^ MSCs prevented the neuronal loss associated with TNBS-induced inflammation. Similarly, MSCs have been shown to prevent neuronal apoptosis [118], increase the survival of motor neurons in amyotrophic lateral sclerosis (ALS) [119, 120] and reduce the loss of dopaminergic neurons in Parkinson’s disease [121]. Furthermore, in a dose-dependent study, 1 × 10^6^ BM-MSCs was optimal to reduce the extent of neural loss in mice with ALS [50].

Enteric neuropathy in intestinal inflammation may be influenced by excessive nitric oxide [122], while inflammation-associated loss of ChAT-IR neurons has been associated with decreases in the number of myenteric neurons [15, 19, 123, 124]. An increase in the total number of nNOS neurons, as well as a decrease in the number of ChAT neurons was revealed 72 hours after the induction of colitis. These results are consistent with previous studies using tissues from IBD patients and experimental animals describing alterations in the neurochemical coding of enteric neurons [16, 23, 24, 125, 126]. In this study, treatment with 1 × 10^5^ MSCs was not effective in attenuating changes in the neurochemical coding of excitatory and inhibitory myenteric neurons. However, the increase in nNOS-IR neurons, as well as the loss of ChAT-IR neurons was attenuated by 1 × 10^6^ and 3 × 10^6^ MSCs. The neurons of the myenteric plexus are primarily responsible for coordinating muscular contraction [14] and prevention of changes to neurochemical coding by MSC treatments has been associated with alleviating TNBS-induced changes to colonic motility [25]. Thus, attenuating changes in the subpopulations of myenteric neurons may alleviate dysmotility associated with intestinal inflammation.

In consistency with our findings, MSCs have been reported to reduce neurological defects and promote functional recovery in experimental models of neurodegenerative diseases [127–131]. While the exact mechanisms of MSC neuroprotection remain unknown, MSCs can act via paracrine mechanisms secreting neuro-regulatory molecules, cytokines, growth factors and chemokines, which provide neuroprotective and neurorestorative effects [132]. These effects include enhancing neuronal viability, promoting regeneration of nerve fibres and inducing the proliferation and differentiation of endogenous neural progenitor cells [133–135]. Furthermore, studies investigating the MSC secretome suggest that numerous bioactive factors secreted by MSCs mediate neuroprotection via tropic support, immunomodulation and anti-apoptosis [134, 135]. The exact MSC-mediated signalling network responsible for neuroprotection of enteric neurons requires further investigation.

In this study, we have observed distinct differences between MSC doses in preventing enteric neuropathy associated with intestinal inflammation. From these results, we can determine that a 1 × 10^5^ dose of BM-MSCs is not adequate, whereas doses of 1 × 10^6^ and 3 × 10^6^ demonstrate anti-inflammatory and neuroprotective qualities in TNBS-induced colitis. Although the 3 × 10^6^ dose MSCs contained triple the quantity of cells than the 1 × 10^6^ dose, no differences were evident between the magnitude of cells homing to and engrafting at the site of tissue injury. This suggests a dose saturation indicating that although there is a greater number of cells being transplanted in vivo, only the required number migrates and engrafts into the inflamed areas of TNBS-induced colitis. This is consistent with a previous MSC study which revealed the engraftment of osteoprogenitor cells to be saturated and concluded that higher doses of cells would be an ineffective strategy to improve engraftment [136]. Furthermore, high-dose inhibition of cytokines has also been observed with high concentrations of MSCs [137–139].

## Conclusions

In this study we have essentially determined an optimal dose of MSCs for enteric neuroprotection in TNBS-induced colitis. We have demonstrated that the neuroprotective and anti-inflammatory effect of BM-MSCs is dose-dependent in TNBS-induced colitis; BM-MSCs have the ability to prevent inflammatory insults to the ENS when administered at a dose of 1 × 10^6^ cells 3 hours after induction of colitis, with no further benefit gained from a higher dose. The findings of this study are important for further investigations into the mechanisms of MSC-based enteric neuroprotection, as well as immunomodulation within the inflamed colon, further enabling MSC therapy to continue to advance forward in future studies.

## Abbreviations

ALS: Amyotrophic lateral sclerosis
BM: Bone marrow
ChAT: Choline acetyltransferase
DMSO: Dimethyl sulfoxide
FBS: Fetal bovine serum
ENS: Enteric nervous system
H&E: haematoxylin and eosin
HLA: Human leucocyte antigen
IBD: Inflammatory bowel disease
IL: Interleukin
IR: Immunoreactive
LMMP: Longitudinal muscle-myenteric plexus
MHC: Major histocompatibility complex
MSC: Mesenchymal stem cell
nNOS: Neuronal nitric oxide synthase
OCT: Optimal cutting temperature
PBS: Phosphate-buffered saline
TNBS: 2,4,6-Trinitrobenzene sulfonic acid
